# Patient Selection for Local Aggressive Treatment in Oligometastatic Non-Small Cell Lung Cancer

**DOI:** 10.3390/cancers13246374

**Published:** 2021-12-19

**Authors:** Raphael S. Werner, Isabelle Opitz

**Affiliations:** Department of Thoracic Surgery, University Hospital Zurich, 8091 Zurich, Switzerland; raphael.werner@usz.ch

**Keywords:** non-small cell lung cancer, oligometastatic, lung cancer surgery, local aggressive therapy

## Abstract

**Simple Summary:**

Since the first introduction of the oligometastatic state with a low burden of metastases in non-small cell lung cancer, accumulating evidence from retrospective and prospective studies has shown that a local aggressive, multimodality treatment may significantly improve the prognosis in these patients. Local aggressive treatment includes a systemic therapy of micrometastatic disease, as well as a radical resection of the primary tumor and surgical resection and/or radiation therapy of distant metastases. However, patient selection and treatment allocation remain a central challenge in oligometastatic disease. In this review, we aimed to address the current evidence on criteria for patient selection for local aggressive treatment in non-small cell lung cancer.

**Abstract:**

One-fourth of all patients with metastatic non-small cell lung cancer presents with a limited number of metastases and relatively low systemic tumor burden. This oligometastatic state with limited systemic tumor burden may be associated with remarkably improved overall and progression-free survival if both primary tumor and metastases are treated radically combined with systemic therapy. This local aggressive therapy (LAT) requires a multidisciplinary approach including medical oncologists, radiation therapists, and thoracic surgeons. A surgical resection of the often advanced primary tumor should be part of the radical treatment whenever feasible. However, patient selection, timing, and a correct treatment allocation for LAT appear to be essential. In this review, we aimed to summarize and discuss the current evidence on patient selection criteria such as characteristics of the primary tumor and metastases, response to neoadjuvant or first-line treatment, molecular characteristics, mediastinal lymph node involvement, and other factors for LAT in oligometastatic NSCLC.

## 1. Introduction

Lung cancer is the most common cause of cancer-related death worldwide and results in more than 36 million disability-adjusted life years globally [1,2]. Unfortunately, lung cancer-related symptoms such as persistent cough, shortness of breath, or chest pain are unspecific and mostly only present in advanced cancer stages. Lung cancer diagnosis is, therefore, often delayed and approximately 70% of all lung cancer diagnoses are made in an advanced stage of the disease [3]. Non-small cell lung cancer (NSCLC) makes up for approximately 85% of all lung cancer cases and itself summarizes a highly heterogeneous group of both histologically, molecularly, and biologically distinct subtypes of lung cancer [4]. In the past years, the gained knowledge about the molecular mechanisms of NSCLC and its immunological microenvironment has driven the development of molecularly targeted therapies and immunotherapy. These approaches have dramatically improved the treatment for patients with NSCLC and provide a prolonged disease control while offering less adverse reactions when compared to the conventional chemotherapy [5,6]. However, despite the emergence of targeted treatment and precision medicine, treatment failure is common and survival in patients with metastatic NSCLC remains poor [7]. The 2-year survival in stage IV disease is estimated between 10% and 23%, and the 5-year survival ranges between 0% and 10% [8]. Nevertheless, stage IV disease is highly heterogeneous and according to the 8th TNM edition, the survival rates may vary widely and are related to the site and number of metastases [8]. In 1995, Hellman and Weichselbaum first described an oligometastatic state of cancer with low systemic tumor burden, few distant metastases, and presumably a less aggressive cancer biology that was associated with an improved survival [9]. In this stage, a metastatic disease might be amenable to local aggressive therapy (LAT), which may include surgical resection of the primary tumor and metastatic lesions and/or a stereotactic body radiation therapy (SBRT). This new concept resulted in a paradigm shift where metastatic NSCLC would not per definition be incurable but require a multi-disciplinary treatment approach to address both the localized primary and metastatic tumor lesions, as well as disseminated, circulating tumor cells [10]. With this approach, the course of the disease may be modified and long-term cancer control may be achieved. However, patient selection and treatment allocation remain a commonly debated and complex topic. In this review article, we aim to present and discuss the current evidence of patient selection criteria for a surgical LAT in oligometastatic NSCLC.

## 2. Definition and Staging of Oligometastatic Non-Small Cell Lung Cancer

Currently, there is no clear consensus on the number of metastatic lesions and number of involved organs that define an oligometastatic state [10]. The majority of published phase II–IV clinical trials on the treatment of oligometastatic NSCLC have used five or fewer metastases in two or fewer organs as a threshold for oligometastasis [11]. Current evidence on LAT in oligometastatic disease is, therefore, limited by the heterogeneous definition and varying inclusion criteria of prospective trials [10]. Due to these controversies, the European Organization of Research and Treatment of Cancer (EORTC) formed a multidisciplinary, pan-European consensus group in 2019 to determine the definition of synchronous oligometastatic NSCLC in a multistep consensus process [12]. The consortium concludes that, in oligometastatic NSCLC, the treatment of all tumor sites should be technically feasible with tolerable toxicity [12]. It was thus proposed that oligometastatic NSCLC should include five or fewer metastases in three or fewer organs [12]. Notably, the primary tumor and an involvement of mediastinal lymph nodes are excluded as a metastatic site, while pulmonary or pleural metastases are counted as a metastatic site [12]. Patients with diffuse serosal metastases (meningeal, pericardial, pleural, or peritoneal) or bone marrow involvement are as well excluded from the definition of oligometastatic NSCLC, for they cannot be treated with radical intent [12]. In general, most (oligo) metastases of NSCLC are found in the brain (35.5%), followed by the contralateral lung (33.6%), the adrenal glands (10%), bones (8.5%), and the liver (2.4%) [10,13]. In addition to the definition of the oligometastatic stage, the EORTC consortium formulated recommendation for the staging work-up. In all patients with suspected oligometastatic disease, 18F-fludeoxyglocose (FDG) positron emission tomography-computed tomography (PET-CT) and brain magnetic resonance imaging (MRI) are recommended. Suspected mediastinal lymph node involvement should be confirmed by either bronchoscopy with endobronchial ultrasound (EBUS) or mediastinoscopy if it is expected to influence the treatment strategy (e.g., to rule out pseudoprogression after immunotherapy or to plan mediastinal irradiation) [12]. Finally, an oligometastatic stage should always be pathologically confirmed by biopsy of at least one metastasis, unless a multidisciplinary team thinks that the risk outweighs the benefit [12]. Especially in contralateral focal ground-glass opacities (GGOs), an EBUS- or CT-guided biopsy and subsequent molecular analysis may help to differentiate between a metastatic process and second primary NSCLC.

## 3. Evidence for Local Aggressive Therapy in Oligometastatic Non-Small Cell Lung Cancer

Since the introduction of the concept of an oligometastatic stage, several retrospective studies have demonstrated that both overall survival (OS) and progression-free survival (PFS) were significantly improved when LAT was applied to all metastatic sites [13,14,15]. These findings were further supported by prospective single-arm clinical studies in oligometastatic NSCLC patients treated with LAT [16,17,18]. However, to date, only two randomized trials have been conducted: Published in 2016, Gomez et al. had randomized 49 patients with non-progressing oligometastatic NSCLC after completing first-line treatment to either palliative maintenance chemotherapy or maintenance chemotherapy and LAT. Since a clear benefit in PFS (3.9 versus 11.9 months) was apparent in patients receiving maintenance chemotherapy and LAT, the study was terminated early [19]. Recent long-term results demonstrated a benefit in OS with a median of 41.2 months in the LAT arm versus a median of 17.2 months in the maintenance chemotherapy arm [20]. In this trial, LAT consisted of hypofractionated radiotherapy or SBRT in 48%, a combination of surgery and radiotherapy in 24%, chemoradiotherapy in 8%, hypofractionated radiotherapy and chemoradiotherapy in 12%, and surgery to all sites in 4% of all cases [19]. In the second randomized trial, Iyengar et al. randomized patients to receive SBRT and maintenance chemotherapy or maintenance chemotherapy alone. After an enrollment of 29 patients, the trial had to be stopped early since an improved median PFS of 9.7 months versus 3.5 months was found [21]. The guidelines of the European Society of Medical Oncology (ESMO) as well as the guidelines of the National Comprehensive Cancer Network (NCCN) recommend a LAT with surgery, SBRT, or definitive radiotherapy in patients with oligometastatic NSCLC [22,23].

## 4. Surgical Treatment for Oligometastatic Non-Small Cell Lung Cancer

**Figure 1:**
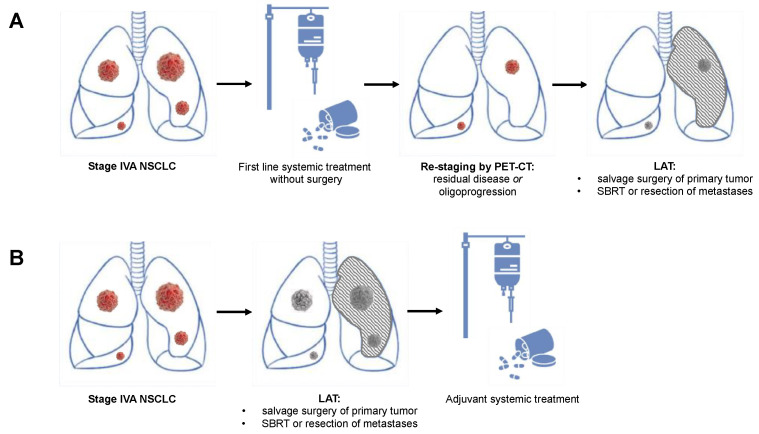
Schematic presentation of the two potential multimodality approaches including local aggressive treatment (LAT) of oligometastatic non-small cell lung cancer with contralateral pulmonary metastases. (**A**) Scheme involving systemic induction treatment, followed by re-staging and LAT with salvage surgery in case of residual disease or oligoprogression. (**B**) Primary LAT with surgical resection or radiation therapy of the primary tumor and all metastatic lesions, followed by adjuvant systemic treatment. LAT: local aggressive therapy.

For LAT of oligometastatic NSCLC, surgical resection has traditionally been the main treatment modality, with more than 50% of all patients receiving surgical treatment in early systematic reviews [24]. Berzenji et al. recently summarized the two most common treatment approaches: The first approach (Figure 1B) includes an initial aggressive resection of the primary tumor, followed by the resection or SBRT of metastatic lesions. Systemic treatment (preferably targeted treatment in NSCLC with targetable oncogenic drivers or immunotherapy in NSCLC without targetable oncogenic drivers but PD-L1 expression >1%) is subsequently used for the control of micrometastatic disease [10]. A second option for addressing oligometastatic NSCLC is a neoadjuvant systemic treatment as described above, followed by a PET-CT-based re-staging and subsequent resection (Figure 1A). In non-progressive or oligoprogressive disease, the resection of the primary tumor and an aggressive treatment of distant metastases by either resection or SBRT follow thereafter in a so-called “salvage” surgery concept [10,25]. Upfront surgery offers the advantage of performing surgery without delay and without the risk of a decline in the functional status after an induction treatment. However, no down-staging is possible and extensive open resections such as pneumonectomies or sleeve resections are often required [26].

**Figure 2:**
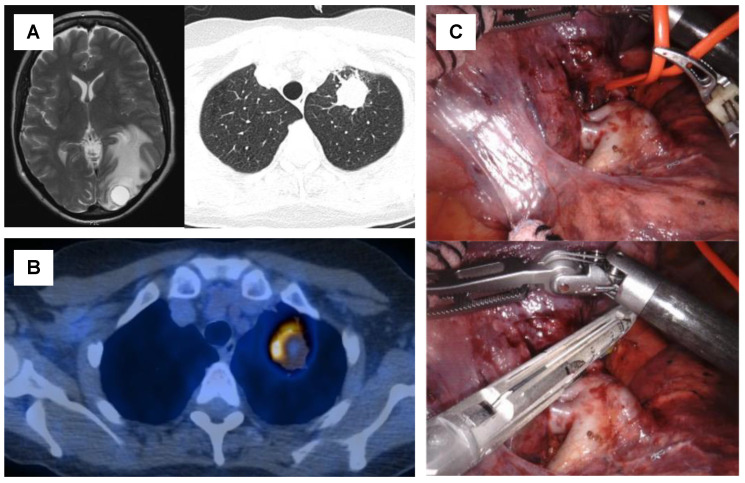
Case of a 57-year-old patient with oligometastatic lung squamous cell carcinoma with a single synchronous brain metastasis and no sign of mediastinal nodal involvement (**A**). The patient underwent surgical resection of the brain metastasis, followed by six cycles of platin-based chemotherapy. Re-staging by PET-CT showed a stable disease of the primary tumor and no signs of additional metastases (**B**). Subsequently, a robotic-assisted thoracoscopic (RATS) upper left lobectomy was performed to complete the local aggressive therapy (LAT) (**C**).

In contrast, neoadjuvant treatment is administered with the intent to eradicate nodal and micrometastatic disease and achieve a reduction in tumor volume and burden, which subsequently enables a complete resection of the remaining tumor (Figure 2) [26]. In addition, neoadjuvant systemic treatment is more likely to provide access for both surgical and systemic treatment modalities to a larger number of patients, while a substantial number of patients may not be able to complete adjuvant treatment if an extensive surgery was performed upfront [26]. Finally, neoadjuvant systemic treatment allows assessing the treatment response and treatment-induced changes in tumor biology on histopathological and molecular levels [26]. This information may provide additional guidance to decide on the further treatment steps. What needs to be considered, however, is that surgery after neoadjuvant treatment may be more challenging than upfront surgery. Especially after combination regimens with chemotherapy and immunotherapy, increased vascular fragility and interstitial exudation, compacted or calcified hilar or mediastinal lymph node stations, and fibrotic changes render surgery in these patients more difficult [27]. However, despite these challenges, even extensive resections in locally advanced stages and after induction with immunotherapy can be safely performed with 90-day mortality rates between 0% and 3% [28,29,30]. The ideal timing of LAT within a multimodality treatment approach is, thus, highly debated and several ongoing clinical trials are currently evaluating different schemes of LAT in combination with targeted therapy, immunotherapy, and/or chemotherapy (Table 1).

Several retrospective cohort studies are reporting promising outcomes of a salvage surgery approach with median OS ranging between 9–75.6 months [31,32,33], 2–5-year survival rates of 20–75% [31,34,35,36,37], and an increased PFS ranging from 5.9 to 43.6 months [31,32,33]. While many of the patients included in these cohorts were treated with conventional chemotherapy, a further increase in OS and PFS is to be expected in patients who underwent neoadjuvant targeted therapy or immunotherapy. A recent study by Ohtaki et al. supports this assumption: In a retrospective cohort of 36 patients who underwent salvage surgery after EGFR- or ALK-TKI treatment, a 3-year OS and PFS of 75.1% and 22.2% were found [38]. A recent retrospective study by Jones et al. additionally supports the concept of a neoadjuvant induction in stage oligometastatic NSCLC by showing that patients who received neoadjuvant therapy had a significantly improved 5-year OS of 40% when compared to the cohort of patients who had received primary surgery (20% 5-year OS) [39]. However, when compared to neoadjuvant chemotherapy followed by local radiotherapy, primary surgery followed by adjuvant chemotherapy still appears to offer an increased median OS (48 months versus 18 months) [40].

The use of pleurectomy and decortication for malignant pleural effusion or disseminated pleural metastases without extrathoracic disease has only been investigated in small sample sizes [41]. Currently, there is no evidence from larger studies and clinical trials to provide a recommendation for LAT in patients with malignant pleural effusion or disseminated pleural metastases [41].

## 5. Radiation Therapy for Oligometastatic Non-Small Cell Lung Cancer

Data on the use of radiation therapy for oligometastatic NSCLC are currently limited, but the majority of the contemporary data supports the use of consolidative SBRT in patients with stable disease or partial response to first-line systemic treatment or in patients with oligoprogression during systemic therapy [42]. Especially in the era of immunotherapy, the combination of SBRT and immune checkpoint inhibitors has been shown to modulate the tumor microenvironment and increase the trigger for a systemic anti-cancer response [42,43]. Most current studies and guidelines recommend an upfront systemic therapy, followed by LAT using SBRT with or without surgery. The American Radium Society currently recommends a cutoff of three metastatic sites or fewer to receive consolidative SBRT [42]. In patients with four to five metastatic lesions, SBRT should be considered on a case-by-case basis. However, the current consensus guidelines are based on smaller phase II trials, while results from larger phase III trials are pending [42]. An enrollment in an ongoing phase 3 trial is, therefore, encouraged when SBRT is planned in patients with oligometastatic NSCLC [42].

## 6. Patient Selection Criteria for Local Aggressive Therapy

Since the evidence of LAT for oligometastatic NSCLC is increasing, the identification and characterization of the patient cohort that will benefit from a LAT strategy has been essential and was highly debated ever since. However, the clinical heterogeneity and broad spectrum of therapeutic approaches make it difficult to identify clear clinical prognostic factors. Here, we discuss a group of prognostic factors that are either associated with the clinical outcome after LAT or are considered to be fundamental for an aggressive treatment of the primary tumor.

### 6.1. Site of the Primary Tumor

In many metastatic NSCLC, the primary tumor is as well locally advanced and may present with an infiltration into the central airways, large vessels, the chest wall, or neurovascular structures, as in pancoast tumors. For a successful LAT, disease control not only concerns the metastatic sites, but also the primary tumor. Current evidence shows that a complete resection of the primary tumor (R0-resection) is critical for the OS and PFS of patients undergoing LAT. R1/R2 resections are associated with a significantly worse OS and PFS than R0 resections in a retrospective analysis of 53 patient [44]. Therefore, extended resections such as sleeve-resections or intrapericardial pneumonectomies should be performed in selected cases if necessary to achieve tumor-free resection margins. Surgical treatment of locally advanced NSCLC may require an intraoperative stand-by or support of extracorporeal membrane oxygenation (ECMO) or cardiopulmonary bypass and may need an experienced postoperative intensive care. Surgery of oligometastatic NSCLC should, therefore, be reserved to expert centers with sufficient case volume. In cases with an unresectable primary tumor, either due to the local extent of the disease or due to a reduced functional capacity, SBRT offers an alternative approach that has been shown to provide favorable local control rates [18,21,45,46]. While in the past, SBRT has mostly been used in situations where surgery was not feasible, recent studies have proven the safety, feasibility, and efficacy of SBRT in oligometastatic NSCLC [18,21,45,46]. However, no clinical trials comparing a surgical approach to SBRT in this setting have been published to date [10].

### 6.2. Site of Metastases

According to the EORTC consensus statement, oligometastatic disease is defined as the stage in which long-term disease control can be gained by LAT. Serosal metastases or bone marrow metastases are, thus, excluded from this definition as they cannot be controlled by local therapy. For solitary metastases in other organs, the size and location as well as the accessibility for surgical resection are essentially influencing the indication for surgical treatment. The decision whether the primary tumor or a metastasis is suitable for surgical resection should always be made in a multidisciplinary tumor board, but it lastly lies in the hands of the surgeon to decide on functional and anatomical feasibility.

The brain is the most common site for distant metastasis in NSCLC and aggressive treatment of cerebral oligometastases including a combination of surgery and radiotherapy is associated with improved OS, improved functional status, and decreased chances for cerebral recurrence [47,48]. Patients with unresectable single brain metastases should be treated with stereotactic radiosurgery or definitive radiotherapy [48]. Similarly, relatively good prognoses have been reported in adrenal oligometastases after radical adrenalectomy [48]. In a study by Raz et al., a median survival of 19 months and 5-year survival of 34% was seen in patients with oligometastatic NSCLC and isolated adrenal metastases undergoing adrenalectomy, whereas patients who were treated without adrenalectomy showed a median survival of 6 months and a 5-year survival of 0%. In particular, patients with ipsilateral adrenal metastases had a significantly improved 5-year survival when compared to contralateral adrenal metastases [49].

### 6.3. Mediastinal Lymph Node Involvement

Mediastinal lymph node involvement has been determined as a predictor for poor prognosis in patients who undergo LAT for oligometastatic NSCLC [15,48,50,51]. Many authors, therefore, conclude that patients with N0 disease are the ideal candidates for LAT, with a 5-year survival up to 21% in patients with synchronous brain metastases and 51% in patients with isolated adrenal metastases [13,48,49,50,51,52]. The role of mediastinal lymph node involvement is further highlighted in the population of oligometastatic NSCLC with extracranial and extra-adrenal metastases. The 5-year survival rate in this population was 64% in patients with N0 status, but 0% in patients with N2 status [51]. Patients with pathologically confirmed N2 disease should, therefore, not be candidates for LAT. In this perspective, we also recommend that suspected lymph node metastases should always be confirmed by bronchoscopy and EBUS or mediastinoscopy in patients with oligometastatic NSCLC. This is especially important in patients who have undergone an induction treatment with immunotherapy and may show pseudoprogression upon restaging by PET-CT [53].

### 6.4. Synchronous and Metachronous Metastases

While synchronous metastases are defined as a manifestation of distant metastases within 6 months of the primary tumor’s diagnosis, metachronous metastases occur more than 6 months after the initial diagnosis [54]. In a large meta-analysis by Ashworth et al., prognostic factors after curative, local, consolidative treatment of oligometastatic NSCLC were evaluated. Metachronous metastasis was a significant predictor for an improved OS (multivariable hazard ratio 3.02) in the cohort and was, therefore, included into a risk classification scheme based on recursive partitioning analysis. The authors describe a low-risk group with metachronous metastases (5-year OS 47.8%), an intermediate-risk group with synchronous metastases and no mediastinal lymph node involvement (5-year OS 36.2%), and a high-risk group presenting with synchronous metastases and N1 or N2 disease (5-year OS 13.8%) [13,54]. Similar findings were reported in a systematic analysis of 114 patients with adrenal metastases of NSCLC. In this cohort, median OS was significantly shorter in synchronous metastases when compared to metachronous metastases (12 months vs. 31 months) [55].

### 6.5. Performance Status

In a multicenter analysis of 124 patients with oligometastatic NSCLC who underwent resection of the primary tumor in Switzerland, a 5-year survival of 83% and a low perioperative morbidity and mortality were found in a subgroup of younger patients (<60 years) with a negative nodal status [15]. These findings fall in line with other studies where patients with a good performance status, aged <65 years, and with solitary metastases survived longer [56,57]. Accordingly, patients who had experienced a weight loss >10% had a significantly decreased median OS of 6 months versus 28 months [44]. The decision for LAT should be made in a multidisciplinary tumor board and functional parameters such as respiratory reserve and a cardiac risk score should, therefore, always be taken into consideration [15]. The improved survival of patients with a good performance status also suggests that this group of patients is more likely to be selected for an aggressive treatment protocol, extensive surgical resection, or a second-line treatment after relapse [15].

### 6.6. Response to Systemic Therapy and Oligoprogressive Disease

The response to the first-line treatment is assumed to be a key prognostic factor for the success of LAT in oligometastatic NSCLC. In contrast to other prospective trials, the single-arm phase II trial by De Ruysscher et al. included 40 patients with oligometastatic NSCLC regardless of their initial response to first-line treatment [18]. The reported median OS of 13.5 months and 5-year survival of 7.7% was considerably worse when compared to results from other prospective trials such as the trials by Gomez et al. (median OS 41.2 months) [18,20]. This discrepancy suggests that LAT should not be performed in patients who show a systemic progression under first-line therapy [54]. However, a special consideration should be given to patients who show a disease progression in one or few sites while under active systemic therapy. In oligoprogressive disease, evidence on the use of LAT is scarce despite the rising number of clinical trials on oligometastatic NSCLC. Nevertheless, current evidence supports the use of LAT on progressing metastatic sites in patients that otherwise responded well to the administered systemic treatment [58]. In a study by Weickhardt et al., an analysis of patients with EGFR- or ALK-mutated NSCLC under tyrosine kinase inhibitor (TKI) therapy showed a successful application of LAT for oligoprogression with a median of more than 6 months of disease control after LAT [59]. Yu et al. retrospectively analyzed 18 patients with oligoprogressive disease under EGFR tyrosine kinase inhibitor (TKI) treatment who underwent LAT including SBRT, radiofrequency ablation, and surgery. Median OS after LAT was 41 months and EGFR-TKI treatment was restarted within 1 month after LAT. The authors, therefore, concluded that LAT is tolerated well in this group of patients and results in favorable OS rates [60]. In addition, both authors highlighted the importance of a continuation of TKI therapy after LAT.

### 6.7. Histopathological and Molecular Markers

To date, only little evidence is present on the association between the histological subtypes, mutational status, or tumor microenvironment and the prognosis of patients with oligometastatic NSCLC undergoing LAT [61]. In the meta-analysis of Ashworth et al., adenocarcinoma histology was associated with an improved OS [13]. However, this association was not supported by other retrospective studies and systematic reviews [15,62]. In a systematic review by Bertolaccini et al., a histologic grading of G1/G2 was found to be a positive prognostic factor for OS [62]. While the molecular mechanisms and the effect of the tumor microenvironment on cancer progression and metastasization have been extensively investigated in model systems, translational studies evaluating the real impact of the tumor microenvironment on treatment success in oligometastatic disease are lacking [61]. However, most of the currently ongoing trials on LAT in oligometastatic NSCLC include a collection of tissue for secondary analyses. In order to deepen the current understanding of the molecular processes in oligometastatic NSCLC, including the intratumoral heterogeneity among different metastases, a collection of tissue samples from the primary tumor as well as the metastatic lesions is essential. Unfortunately, only one trial (NCT03827577) requires the collection of tissue from the primary tumor as well as from the resected oligometastases [61].

To date, a number of blood-based molecular biomarkers have been investigated in oligometastatic NSCLC [54]. In a multivariable analysis by Ohtaki et al., high preoperative levels of carcinoembryonic antigen (CEA) were an independent prognostic factor for OS [38]. Patients with preoperative CEA levels below 5 ng/mL showed a 3-year OS of 84.0%, whereas patients with preoperative CEA levels above 5 ng/mL had a 3-year OS of 50.8% [38]. In addition, an analysis of microRNA expression profiles identified a unique upregulation of members of the microRNA-200 family in tissue from oligometastatic patients who progress to polymetastases compared to those who remain oligometastatic [63]. The same pattern was recreated in a NSCLC xenograft model [63,64]. The distinct microRNA patterns were found in tissue from histologically different tumors, suggesting a common molecular basis for an oligometastatic state [63]. In the future, these “oligoMirs” may help to identify patients with an oligometastatic progression with increased accuracy [65]. With the ongoing collection of blood in the current clinical trials, more evidence on blood-based biomarkers such as circulating tumor DNA or micro RNA can be expected and may help to generate new prognostic and predictive scores to guide LAT and precision medicine in oligometastatic NSCLC [61].

### 6.8. Quality of Life

Patients scheduled for LAT are facing a long course of treatment including a multitude of different treatment modalities. In these patients, not only a regular monitoring of the physical functional status but also of the patient’s quality of life is essential. Patient-reported outcome measures (PROMs) are defined PROMs as the patient’s subjective perception of physical, psychological, social, and somatic functioning and overall well-being [66]. PROMs are, therefore, intended to monitor the patient’s perception of his or her general health status and well-being during the treatment of diseases such as metastatic NSCLC. PROMs thereby provide important information that can complement traditional clinical outcomes used in medical care and are being established as an important tool in understanding patients’ perceptions of their symptoms and their global health status [67]. For lung cancer patients, the EORTC QLQ-C30 and its lung cancer-specific module QLQ-LC13 [68] are most frequently used and often supplemented by EQ-5D-5L [69] for health-economic evaluation to calculate quality-adjusted life years [70,71,72]. Despite their importance for assessing the patients’ wellbeing, PROMs have not yet been evaluated in prospective trials for LAT in oligometastatic NSCLC and remain to be investigated in future clinical trials.

## 7. Conclusions and Outlook

With the recent innovations in treatment strategies, such as targeted-treatment-based or immunotherapy-based combinations, future multimodal treatment concepts are expected to become more personalized and precise. Additional evidence on molecular characteristics including mutational status, immune microenvironment, and tumor spatial biology will be provided by future analyses and clinical trials. These data may help to further personalize the allocation to different treatment protocols and sequences. Early advances in organoid-based ex vivo chemosensitivity assays have shown promising results and, although a systematical clinical application is currently not possible, organoid-based drug screening may help to select the ideal systemic treatment in the future [73]. In general, the establishment of personalized risk classification groups according to functional, histological, molecular, or radiological information such as total tumor volume, mutational status, or pulmonary function would, therefore, be of great interest. However, the current lack of data in this field does not yet allow for the generation of detailed risk classification groups among oligometastatic NSCLC and it remains to be evaluated how the treatment of oligometastatic NSCLC should be tailored and personalized to different risk groups.

In future trials, the addition of new systemic treatment such as targeted agents and immunotherapy may lead to a further improvement of the outcomes after LAT, especially since most historical cohorts were based on treatment protocols that only included conventional chemotherapy.

Since both the diagnostic patient work-up and the treatment of oligometastatic NSCLC require a multidisciplinary team including oncologists, radiation therapists, thoracic surgeons and neurosurgeons, pulmonologists, radiologists, anesthesiologists, and intensivists, the treatment should only be performed in expert centers with sufficient case volume.

## Figures and Tables

**Table 1:** Ongoing clinical trials for local aggressive therapy including surgery in oligometastatic non-small cell lung cancer. LAT: local aggressive treatment. OPD: oligoprogressive disease. OS: overall survival. PFS: progression-free survival. SBRT: stereotactic body radiation therapy.

Study Abbreviation	ClinicalTrails.gov Identifier	Phase	Setting	Type of Systemic Treatment	Type of LAT	Timing of LAT	n	No. of Metastases	Primary End Points	Planned Completion
14-18 CHESS	NCT03965468	II	Synchronous oligometastatic NSCLC	Durvalumab, Carboplatin, Paclitaxel	Primary: Surgery or radical radiotherapyMetastases: SBRT	Neoadjuvant systemic treatment	47	Max. 3	PFS	12/2021
OMEGA	NCT03827577	III	Oligometastatic NSCLC	Standard medical therapy	Surgery, Radiotherapy, RFA	Neoadjuvant systemic treatment or primary LAT	195	Max. 3	OS	09/2022
n/a	NCT02759835	II	EGFR-mutated OPD NSCLC	Osimertinib	Surgery, SBRT, radiofrequency ablation	LAT after oligoprogression under first-lineOsimertinib	37	n/a	PFS	09/2022
n/a	NCT02316002	II	Oligometastatic NSCLC	Adjuvant Pembrolizumab	Completed first-line treatment (surgery, SBRT, radiotherapy, chemotherapy)	Any first-line treatment followed by adjuvant pembrolizumab	51	n/a	PFS	09/2022
LONESTAR	NCT03391869	III	Stage IV NSCLC (incl. OMD subgroup)	Nivolumab and ipilimumab	Surgery, radiotherapy	Combined neoadjuvant and adjuvant immunotherapy	270	n/a	OS	12/2022
NORTHSTAR	NCT03410043	II	EGFR-mutatedStage IIIB or IV NSCLC (incl. OMD subgroup)	Osimertinib	Surgery, radiotherapy	Combined neoadjuvant and adjuvant Osimertinib	143	n/a	PFS	01/2023
LAT-FLOSI	NCT04216121	IIb	EGFR-mutated OPD NSCLC	Osimertinib	Surgery, SBRT	LAT after oligoprogression under first-lineOsimertinib	39	Max. 3	PFS	08/2023
